# Homocysteine Level Is Associated with White Matter Hyperintensity Locations in Patients with Acute Ischemic Stroke

**DOI:** 10.1371/journal.pone.0144431

**Published:** 2015-12-07

**Authors:** Yuan Gao, Sen Wei, Bo Song, Jie Qin, Hui Fang, Yan Ji, Rui Zhang, Shilei Sun, Yuming Xu

**Affiliations:** Department of Neurology, the First Affiliated Hospital of Zhengzhou University at Zhengzhou, Henan, China; Shanghai Institute of Hypertension, CHINA

## Abstract

**Background and Purpose:**

The relationship between plasma level of total homocysteine (tHcy) and white matter hyperintensities (WMHs), especially in patients with acute ischemic stroke (AIS), is controversial. The present study investigated the association between these two as well as WMH locations in a large cohort of patients with AIS.

**Methods:**

Consecutive patients were reviewed from a prospective ischemic stroke database. Clinical data, including tHcy level and WMHs, were assessed. WMHs were assessed using the Fazekas scale and Age-Related White Matter Changes (ARWMC) visual grading scale. The association between tHcy and WMH locations was investigated by using multivariate logistic regression analyses.

**Results:**

A total of 923 out of 1,205 patients were examined. The average age was 58.9 ± 11.9 years; 31.6% were female. Elevated tHcy level was significantly associated with WMHs. For the highest tHcy quartile, the odds ratio (OR) (95% confidence interval; CI) was 1.891 (1.257; 2.843) according to the Fazekas scale and 1.781 (1.185; 2.767) according to the ARWMC scale when compared to the lowest quartile. However, in a subgroup analysis, only WMHs in the periventricular area and left or right frontal areas were found to be independently associated with tHcy level. For the highest tHcy quartile, the OR (95% CI) was 1.761 (1.172; 2.648) for the periventricular WMHs, 1.768 (1.134; 2.756)for the left frontal WMHs, and 1.890 (1.206; 2.960)for the right frontal WMHs.

**Conclusions:**

In patients with AIS, plasma tHcy level is related to WMHs, especially WMHs distributed within the periventricular and frontal areas.

## Introduction

Cerebral white matter hyperintensities (WMHs) are areas of hypointense signals on T1-weighted images and hyperintense signals on T2-weighted and fluid-attenuated inversion recovery (FLAIR) images obtained from magnetic resonance imaging (MRI) of the brain [1]. Despite the fact that vascular risk factors, such as age, hypertension, and diabetes mellitus, are related to WMHs [2], the underlying pathological mechanisms are still poorly understood [3]. Expanding studies suggest that plasma total homocysteine (tHcy) level is associated with WMHs in general populations [4] and even in stroke patient [5]; however, other studies have failed to replicate this association [6]. Very few studies have investigated the correlation between elevated tHcy level and WMH locations [7,8] and these studies produce conflicting results. Some have reported that tHcy is related to deep rather than periventricular WMHs (PWMHs) [9], while others do not show a significant relationship between tHcy and deep WMHs (DWMHs). Even fewer studies have examined the association between tHcy level and WMHs in different lobes [10], and no study has examined it in patients with acute ischemic stroke (AIS). Thus, the present study included a large sample of patients with AIS with the goal of determining the association between plasma tHcy levels and WMHs as well as WMH locations.

## Materials and Methods

### Study population and data collection

Patients were consecutively selected from a prospectively collected ischemic stroke database from the First Affiliated Hospital of Zhengzhou University (Henan, China). All consenting patients were included from January 2012 to December 2014, with the following inclusion criteria: (1) age ≥ 18 years; (2) hospitalized with a primary diagnosis of acute ischemic stroke according to the World Health Organization’s criteria and confirmed by a brain MRI within 14 days after stroke onset; and (3) no severe physical illnesses that could be life threatening. Patients with hemorrhagic stroke or silent stroke (e.g., without signs or symptoms) or those who did not have MRI scans or homocysteine measurements were excluded.

### Ethics Statement

This study was approved by the Ethics Committee of the First Affiliated Hospital of Zhengzhou University. All patients or their legally authorized representatives signed an informed consent form.

### Data collection

The National Institutes of Health Stroke Scale (NIHSS) scores were used for evaluating stroke severity. Systolic blood pressure (SBP) and diastolic blood pressure (DBP) on admission were also measured. Risk factors based on past medical history ascertained via direct patient and/or proxy interviews were coded as follows: history of tobacco use was defined as current or past tobacco use; history of alcohol use was defined as current or past alcohol intake; hypertension was defined as a previous record (on different days before stroke onset) of at least 2 raised blood pressure measurements of either ≥140 mm Hg systolic or ≥ 90 mm Hg diastolic, the use of antihypertensive medications, or a physician’s diagnosis. Stroke history was defined as a medically confirmed history of an ischemic or hemorrhagic stroke or subarachnoid hemorrhage. Coronary artery disease (CAD) was defined as a history of angina pectoris, myocardial infarction, or using CAD medication. Atrial fibrillation (AF) was defined as a history of AF or using AF medication. Diabetes was defined as a 2-hour oral glucose tolerance test value of ≥ 200 mg/dL, insulin or oral hypoglycemic use, or a physician’s diagnosis. Hyperlipidemia was defined as a history of hyperlipidemia, using lipid-lowering medications, or a physician’s diagnosis.

### Blood measurements

Fasting blood samples were collected in evacuated tubes containing EDTA, after an overnight fasting of at least 8 hours, and centrifuged within 1 h and stored below -20°C until analyzed. Total homocysteine level was measured using a florescence polarization immunoassay analyzer (Abbott Laboratories, Chicago, IL).

### WMH imaging

T2-weighted FLAIR sequence and diffusion weighted imaging (DWI) of brain MRI scans was used to assess WMHs, which were analyzed by two neurologists who were blinded to the clinical data. Disagreements were resolved through consensus. The Fazekas scale [11] and Age-Related White Matter Changes (ARWMC) [12] visual grading scale were used for rating WMHs. According to the Fazekas scale, the WMHs were divided into PWMHs graded as absent (grade 0), cap (grade 1), smooth halo (grade 2), or irregular and extending into the subcortical white matter (grade 3), and DWMHs were graded as absent (grade 0), punctate foci (grade 1), early-confluent (grade 2), or confluent (grade 3). The final white matter lesion severity is the sum of the two regions ranging from 0–6. According to the ARWMC measure using a 4-point scale, the following 5 regions in the right and left hemispheres were respectively analyzed: the frontal area, the parieto-occipital area, temporal area, basal ganglia, and infratentorial area [12]. Basic scores for each brain area were determined, and the sum of scores for all brain areas was used as a measure of lesion severity. The two above-mentioned scales both assessed WMH severity and locations. After being tested on a set of images from 80 consecutive patients, inter-rater reliabilities between the 2 investigators were 0.89 and 0.88 for the total Fazekas and ARWMC scales, respectively.

### Statistical analyses

The summed WMH scales were stratified into mild WMHs (Fazekas score, 0–2; ARWMC score, 0–4) and severe WMHs (Fazekas score, > 2; ARWMC score, > 4). WMHs in each element of the Fazekas and ARWMC scales were categorized into mild (0–1) and severe (2–3), respectively. Total homocysteine concentrations were quartered: < 13.0 μmol/L, 13.0–16.5 μmol/L, 16.5–22.13 μmol/L, and > 22.13 μmol/L. The lowest quartile was set as a reference. Estimated glomerular filtration rate (eGFR), an indicator of baseline kidney function, was calculated with the Modification of Diet in Renal Disease formula [13].

All categorical variables were reported as a proportion/percentage of the total, while all continuous variables were summarized as a mean value (±standard deviation (SD)) or a median value with an interquartile range (IQR). The associations between baseline characteristics and WMH severity were tested using the Mann–Whitney U test for continuous variables and χ2 statistics for categorical variables. All factors associated with WMHs from the univariate analyses at a threshold of p < 0.1 were included in the multivariate model as candidate variables and removed via a forward selection procedure. All statistical analyses were performed with SPSS version 19.0. (SPSS, Inc., Chicago, IL, USA). Two-tailed tests with a probability of p < 0.05 were used to estimate statistical significance for all analyses.

## Results

### Study population and baseline characteristics

A total of 1,205 patients with AIS were consecutively registered. A total of 201 patients without MRI images were excluded, as well as 81 patients with no tHcy measurements. The remaining 923 patients comprised the study population. There were no significant differences in terms of baseline clinical and laboratory characteristics between the patients included (n = 923) and those excluded (n = 282). Demographic and clinical characteristics are presented in Table 1.

**Table 1:** Demographic and clinical characteristics.

characteristics	Total N = 923
**Sex(female), n (%)**	**292(31.6)**
**Age, years** *	**58.9(11.9)**
**Tobacco use, n (%)**	**271(29.4)**
**Alcohol use, n (%)**	**212(23.0)**
**Hypertension, n (%)**	**549(59.5)**
**Prior stroke, n (%)**	**226(24.5)**
**Coronary artery disease, n (%)**	**94(10.2)**
**Atrial fibrillation, n (%)**	**19(2.1)**
**Diabetes mellitus, n (%)**	**199(21.6)**
**Dyslipidemia, n (%)**	**65(7.0)**
**NIHSS**	**3(2, 5)**
**SBP, mmHg**	**140(130,154)**
**DBP, mmHg**	**85(78, 92)**
**Haemoglobin, g/L**	**135(126, 145)**
**Hematocrit, %**	**41(38, 43.8)**
**FPG, mmol/L**	**5.2(4.6, 6.4)**
**Creatinine, umol/L**	**67(57, 78)**
**eGFR, ml/min/1.73 m** ^**2**^	**105.05(89.74, 122.22)**
**Uric acid, umol/L**	**276(219, 329)**
**Triglyceride, mmol/L**	**1.36(0.98, 2.03)**
**Total cholesterol, mmol/L**	**4.20(3.58, 4.90)**
**HDL-C, mmol/L**	**1.03(0.88, 1.25)**
**LDL-C, mmol/L**	**2.61(2.07, 3.23)**
**tHcy, μmol/L**	**16.50(13.00, 22.13)**
**Fazekas scale**	
**mild, n (n%)**	**458 (49.6)**
**severe, n (n%)**	**465 (50.4)**
**ARWMC score**	
**mild, n (n%)**	**535 (58.0)**
**severe, n (n%)**	**388 (42.0)**

Values are median (interquartile range) or n (%) unless otherwise stated.

*Mean (standard deviation)

**Abbreviations:** NIHSS, National Institutes of Health Stroke Scale; SBP, Systolic blood pressure; DBP, diastolic blood pressure; FPG, Fasting plasma glucose; eGFR, estimated glomerular filtration rate; HDL-C, high density lipoprotein cholesterol; LDL-C, low density lipoprotein cholesterol; tHcy, total homocysteine; ARWMC, Age-Related White Matter Changes.

### Total homocysteine and WMH severity

In the univariate analysis (Table A in S1 Table), tHcy, age, systolic blood pressure (SBP), alcohol use, hypertension, prior stroke, haemoglobin, hematocrit, estimated glomerular filtration rate (eGFR), triglyceride (TG) and HDL-C (all p<0.1) were associated with WMH severity according to the Fazekas scale, while tHcy, age, SBP, diastolic blood pressure (DBP), hypertension, prior stroke, coronary artery disease (CAD), creatinine, eGFR, TG and HDL-C (all p<0.1) were associated with WMH severity according to the ARWMC scale. In the multivariate logistic regression analysis, tHcy was independently related to WMH severity after adjusting for those confounders. For the highest tHcy quartile, the odds ratios (OR; 95% confidence interval; CI) were 1.891(1.257; 2.843) according to the Fazekas scale and 1.781(1.185; 2.767) according to the ARWMC scale (Table 2).

**Table 2:** The associations of total homocysteine (quartiles) with WMH severity.

	Fazekas score†		ARWMC score‡	
	OR (95% CI)	P value	OR (95% CI)	P value
**Age**	**1.072(1.057, 1.087)**	**<0.001**	**1.061(1.046, 1.075)**	**<0.001**
**Hypertension**	**2.049(1.520,; 2.762)**	**<0.001**	**1.935(1.432; 2.615)**	**<0.001**
**Prior stroke**	**2.296(1.620; 3.254)**	**<0.001**	**2.533(1.813; 3.538)**	**<0.001**
tHcy(μmol/L)*		**0.009**		**0.022**
**Quartile 1**	**Reference**	**---**	**Reference**	**---**
**Quartile 2**	**1.635(1.090, 2.451)**	**0.017**	**1.226(0.815; 1.845)**	**0.329**
**Quartile 3**	**1.766(1.176; 2.654)**	**0.006**	**1.616(1.076; 2.428)**	**0.021**
**Quartile 4**	**1.891(1.257; 2.843)**	**0.002**	**1.781(1.185; 2.676)**	**0.005**

^†^ Included covariables:age, hypertension, prior stroke, alcohol use, systolic blood pressure, haemoglobin, hematocrit, estimated glomerular filtration rate, triglyceride, high density lipoprotein cholesterol and total homocysteine.

^‡^ Included covariables:age, hypertension, prior stroke, coronary artery disease, Systolic blood pressure, haemoglobin, creatinine, estimated glomerular filtration rate, triglyceride, high density lipoprotein cholesterol and total homocysteine.

*tHcy levele was divided by quartiles: quartile 1, tHcy < 13.0μmol/L; quartile 2, tHcy 13.0 to 16.5 μmol/L; quartile 3, tHcy 16.5 to 22.13 μmol/L; quartile 4, tHcy > 22.13 μmol/L. The lowest quartile was set as a reference, the other quartiles are compared to the lowest quartile.

**Abbreviations:** ARWMC, Age-Related White Matter Changes; tHcy, total homocysteine.

### Total homocysteine and WMH locations

In the univariate analysis (Tables B and C in S1 Table) for the Fazekas scale, tHcy was significantly associated with PWMHs rather than DWMHs. In addition, age, SBP, hypertension, prior stroke, haemoglobin, hematocrit, eGFR, TG and HDL-C (all p<0.1) were associated with PWMHs. After adjusting for those confounders, the association between tHcy and PWMHs was still significant. For the highest tHcy quartile, the OR (95% CI) was 1.761(1.172; 2.648) (Table 3).

**Table 3:** The associations of total homocysteine (quartiles) with PWMHs and DWMHs.

	PWMHs†		DWMHs‡	
	OR (95% CI)	P value	OR (95% CI)	P value
**Age**	**1.072(1.058;1.088)**	**<0.001**	**1.042(1.025; 1.059)**	**<0.001**
**Hypertension**	**1.948(1.443; 2.629)**	**<0.001**	**---**	**NS**
**Prior stroke**	**2.187(1.556; 3.076)**	**<0.001**	**1.588(1.077; 2.343)**	**0..020**
tHcy(μmol/L)*		**0.035**		**NS**
**Quartile 1**	**Reference**	**---**	**Reference**	**---**
**Quartile 2**	**1.530(1.020; 2.295)**	**0.040**	**---**	**NS**
**Quartile 3**	**1.581(1.053; 2.347)**	**0.027**	**---**	**NS**
**Quartile 4**	**1.761(1.172; 2.648)**	**0.007**	**---**	**NS**

^†^ Included covariables:age, hypertension, prior stroke, Systolic blood pressure, haemoglobin, hematocrit, estimated glomerular filtration rate, triglyceride high density lipoprotein cholesterol and total homocysteine.

^‡^ Included covariables:age, hypertension, prior stroke, alcohol use, Systolic blood pressure, diastolic blood pressure, haemoglobin, fasting plasma glucose, estimated glomerular filtration rate.

*tHcy levele was divided by quartiles: quartile 1, tHcy < 13.0μmol/L; quartile 2, tHcy 13.0 to 16.5 μmol/L; quartile 3, tHcy 16.5 to 22.13 μmol/L; quartile 4, tHcy > 22.13 μmol/L. The lowest quartile was set as a reference, the other quartiles are compared to the lowest quartile.

**Abbreviations:** PWMHs, periventricular white matter hyperintensities; DWMHs, deep white matter hyperintensities; ARWMC, age-related white matter changes; tHcy, total homocysteine; NS, no significance.

Analyses also observed that tHcy was related to WMHs in the left frontal, right frontal, and left parieto-occipital areas. However, based on a multivariate logistic regression analysis, an association was only revealed in the left frontal and right frontal WMHs. For the highest tHcy quartile, ORs (95% CI) were 1.768(1.134; 2.756)and1.890(1.206; 2.960), respectively (table 4).

**Table 4:** The associations of total homocysteine (quartiles) with the frontal WMHs.

	Left frontal area†		Right frontal area‡	
	OR (95% CI)	P value	OR (95% CI)	P value
**Age**	**1.078(1.060; 1.095)**	**<0.001**	**1.065(1.049; 1.081)**	**<0.001**
**Hypertension**	**1.700(1.209; 2.392)**	**0.002**	**1.643(1.175; 2.296)**	**0.004**
**Prior stroke**	**1.711(1.211; 2.417)**	**0.002**	**1.810(1.282; 2.556)**	**0.001**
tHcy(μmol/L)*		**0.045**		**0.032**
**Quartile 1**	**Reference**	**---**	**Reference**	**---**
**Quartile 2**	**1.145(0.727; 1.805)**	**0.559**	**1.331(0.840; 2.108)**	**0.224**
**Quartile 3**	**1.535(0.983; 2.396)**	**0.060**	**1.666(1.060; 2.618)**	**0.027**
**Quartile 4**	**1.768(1.134; 2.756)**	**0.012**	**1.890(1.206; 2.960)**	**0.005**

^†^ Included covariables:age, hypertension, prior stroke, coronary artery disease, Systolic blood pressure, diastolic blood pressure, haemoglobin, estimated glomerular filtration rate, triglyceride and total homocysteine.

^‡^ Included covariables:age, hypertension, prior stroke, dyslipidemia, Systolic blood pressure, haemoglobin, creatinine, estimated glomerular filtration rate, total cholesterol, low density lipoprotein cholesterol and total homocysteine.

*tHcy levele was divided by quartiles: quartile 1, tHcy < 13.0μmol/L; quartile 2, tHcy 13.0 to 16.5 μmol/L; quartile 3, tHcy 16.5 to 22.13 μmol/L; quartile 4, tHcy > 22.13 μmol/L. The lowest quartile was set as a reference, the other quartiles are compared to the lowest quartile.

**Abbreviations:** tHcy, total homocysteine.

## Discussion

The present study demonstrates that plasma tHcy level is not only related to WMHs but also WMH locations, which are distributed within the periventricular and frontal areas in patients with AIS.

As an image marker of small-vessel disease, WMHs are often considered to involve small intracerebral vessel damage, resulting in chronic or recurrent hypoperfusion [14]. Several studies have shown that homocysteine can activate a series of complex processes, including the promotion of coagulation [1], decreasing arterial reactivity to vasomotor stimuli [15], activating platelets [16], increasing the production of free oxygen radicals [17], and stimulating proliferation of smooth muscle cells in the arterial wall [18] leading to small intracerebral vessel damage. The fact that tHcy concentration is higher in patients with lacunar strokes than other stroke types [17,18] suggests a selective sensitivity among small cerebral arteries to the effects of Hcy.The present results indicate that tHcy may contribute to processes underlying WMHs in patients with AIS, which is consistent with previous studies [5]. Surprisingly, we also found that tHcy level was related more to PWMHs rather than DWMHs. This result could be due to PWMHs perhaps having a divergent pathogenesis from DWMHs. The former often relates to myelin pallor or rarefaction without other convincing evidence for ischemia, while the latter is likely to be induced by ischemic etiology, as suggested by MRI histopathological correlative studies [19]. Although Sachdev et al. concluded that high tHcy levels were related to increased DWMHS, but not PWMHs, in a healthy community sample, the relationship was only significant in men [9]. A perfusion study found that DWMHs were associated with reduced cerebral blood flow; however, post-mortem examinations showed no ischemic changes in DWMHs; rather, a severe loss of myelin and astrocytic gliosis was observed [20]. Furthermore, a population-based autopsy study supports the association between tHcy level and PWMHs [7], which is consistent with results from the present study. Therefore, the association between tHcy level and PWMHs might be a direct consequence of subsequent axonal loss in fiber tracts running near the lateral ventricles or an indirect consequence of subsequent ventricular dilatation and CSF leakage [7].

Interestingly, the present study also found that tHcy was related to frontal WMHs, which was rarely reported in patients with AIS. Differential frontal lobe vulnerability to vascular risks has been well documented [21]. Unlike in the other lobes, almost all middle-aged and older healthy adults have at least some WMHs in the frontal lobes [21,22], and the longitudinal rate of WMH expansion is faster than in other lobes [23]. In acute ischemic stroke, the frontal regions experience the worst damage [24]. There are findings regarding differential effects of age and vascular risk factors on temporal and parietal WMHs as well; thus, differences between the frontal lobes and other brain regions are likely reflective of degree rather than kind. However, the frontal lobes appear to be most consistently affected [10]. As a vascular factor, tHcy had a selective vulnerability on frontal WMHs through the above mechanisms in the current study. As reported in previous studies, tHcy is associated with cognitive impairment [5,25]. The association between frontal WMHs and cognitive impairment has also been demonstrated [26]. Therefore, we hypothesized that tHcy might influence cognitive functioning by contributing to WMH processing in the frontal area. Future studies are needed to examine this hypothesis,

Strengths of the present study are as follows. Firstly, a large sample of patients with acute ischemic stroke was included. Secondly, all patients underwent 3.0T MRI scans, which was advantageous due to its high spatial resolution and sufficient detection of small WMHs. Thirdly, two different visual rating scales were combined to evaluate WMH severity and locations based on both T2-weighted fluid attenuated inversion recovery (FLAIR) sequences and diffusion weighted imaging (DWI) parameters. This allowed differentiation between WMHs and acute/subacute ischemic lesions. Comprehensive information obtained from each patient ensured a clearer examination of risk factors and results.

Some limitations should also be noted. Firstly, the current study utilized a cross-sectional design; thus, a causal relationship between tHcy and the degree and distribution of WMHs needs further confirmation using a longitudinal study. Secondly, it has been recognized that folate and vitamin B12 deficiency could lead to variations in Hcy levels [27]. The effects of these vitamins on Hcy variations was not observed in the present study due a lack of serum folate and vitamin B12 screenings, and patients were not provided folate and vitamin B supplements before their assessments. Even in cases where patients have been supplied with folate and vitamin B12, their effects take a few weeks to have any significant influence on tHcy concentrations [28]. Thirdly, there is no examination of gene polymorphism in the present study. Several reports demonstrate the MTHFR gene is involved in plasma Hcy levels and may contribute to endothelial dysfunction, which is one of the suggested mechanisms behind WMH [29]. Some studies suggest a trend for association between the MTHFR and WMH [30]. Future studies will be involved this aspect. Last but not least, the visual rating scales assessing WMH severity and locations were semi-quantitative rather than quantitative. Although possibly less sensitive and accurate, the two scales have been widely used, are robust, and are easy to perform in everyday clinical practice. Previous studies have shown that visual rating scales adequately reflect actual WMH volumes [31].

In conclusion, the present study revealed that plasma tHcy is not only related to WMHs but also to WMH locations. These locations are distributed within the periventricular and frontal areas in patients with AIS.

## Supporting Information

S1 File Supporting information file (Primary data). (XLS)

S1 Table Supplemental Tables. (DOCX)
