# Supplementary material for: Homocysteine Level Is Associated with White Matter Hyperintensity Locations in Patients with Acute Ischemic Stroke
Source: PLoS One. 2015 Dec 7;10(12):e0144431. doi: 10.1371/journal.pone.0144431 (PMC4671668; doi:10.1371/journal.pone.0144431)
Supplement: S1 Table — (DOCX) [file pone.0144431.s002.docx]

**Table A. Univariate analysis for predictors of WMH severity**

|  | **Fazekas scale** | | | **ARWMC scale** | | |
| --- | --- | --- | --- | --- | --- | --- |
|  | **mild** | **severe** | **p-Value** | **Mild** | **severe** | **p-Value** |
| **Sex(female), n (%)** | **136(29.7)** | **156(33.5)** | **0.208** | **166(31.0)** | **126(32.5)** | **0.641** |
| **Age(years)** | **55(47, 62)** | **63(57, 76)** | **<0.001** | **57(48, 63)** | **63(57, 70)** | **<0.001** |
| **SBP(mmHg)** | **139(126.0, 150.0)** | **142(130.0, 156.5)** | **<0.001** | **139.0(127.0, 150.0)** | **142(130, 159)** | **<0.001** |
| **DBP(mmHg)** | **85(76, 93)** | **85(78, 90)** | **0.625** | **84(77, 92)** | **86(79, 92)** | **0.165** |
| **NIHSS score( points)** | **3(1, 5)** | **3(2, 6)** | **0.220** | **3(1, 5)** | **3(2, 6)** | **0.405** |
| **Tobacco use, n (%)** | **145(31.7)** | **126(27.1)** | **0.128** | **165(30.8)** | **106(27.3)** | **0.246** |
| **Alcohol use, n (%)** | **116(25.3)** | **96(20.6)** | **0.091** | **132(24.7)** | **80(20.6)** | **0.148** |
| **Hypertension, n (%)** | **226(49.3)** | **323(69.5)** | **<0.001** | **275(51.4)** | **274(70.6)** | **<0.001** |
| **Prior stroke, n (%)** | **70(15.3)** | **156(33.5)** | **<0.001** | **85(15.9)** | **141(36.3)** | **<0.001** |
| **CHD, n (%)** | **40(8.7)** | **54(11.6)** | **0.148** | **47(8.8)** | **47(12.1)** | **0.099** |
| **Atrial fibrillation, n (%)** | **9(2.0)** | **10(2.2)** | **0.843** | **12(2.2)** | **7(1.8)** | **0.643** |
| **Diabetes mellitus, n (%)** | **102(22.3)** | **97(20.9)** | **0.602** | **113(21.1)** | **86(22.2)** | **0.704** |
| **Dyslipidemia, n (%)** | **35(7.6)** | **30(6.5)** | **0.480** | **42(7.9)** | **23(5.9)** | **0.260** |
| **Haemoglobin(g/L)** | **137(127, 146)** | **134(125, 143)** | **0.003** | **137(127, 146)** | **134(125, 143)** | **0.021** |
| **Hematocrit(%)** | **41.4(38.0, 44.0)** | **40.6(37.7, 43.2)** | **0.012** | **41.1(38.0, 44.0)** | **40.9(37.6, 43.4)** | **0.069** |
| **FPG(mmol/L)** | **5.2(4.6, 6.5)** | **5.2(4.7, 6.4)** | **0.889** | **5.2(4.6, 6.4)** | **5.2(4.7, 6.4)** | **0.301** |
| **Creatinine(μmol/L)** | **66(56, 77)** | **68(57, 78)** | **0.091** | **66(55, 77)** | **68(59, 79)** | **0.019** |
| **eGFR(ml/min/1.73 m²)** | **108.9(92.9, 129.2)** | **101.6(86.5, 115.8)** | **<0.001** | **108.6(92.2, 127.8)** | **101.1(86.4, 115.7)** | **<0.001** |
| **Uric acid(μmol/L)** | **279(222.8, 329.3)** | **275(216.5, 328.0)** | **0.296** | **273(218, 328)** | **280(219.3, 332.0)** | **0.378** |
| **Triglyceride (mmol/L)** | **1.5(1.0, 2.2)** | **1.3(1.0, 1.8)** | **0.001** | **1.4(1.0, 2.1)** | **1.3(1.0, 1.9)** | **0.023** |
| **Total cholesterol (mmol/L)** | **4.2(3.6, 4.9)** | **4.2(3.6, 4.9)** | **0.765** | **4.2(3.6, 4.9)** | **4.1(3.6, 4.9)** | **0.556** |
| **HDL-C(mmol/L)** | **1.0(0.9, 1.2)** | **1.1(0.9, 1.3)** | **0.003** | **1.0(0.9, 1.2)** | **1.1(0.9, 1.3)** | **0.008** |
| **LDL-C(mmol/L)** | **2.6(2.1, 3.3)** | **2.6(2.1, 3.2)** | **0.738** | **2.6(2.1, 3.3)** | **2.6(2.0, 3.1)** | **0.211** |
| **tHcy(>22.13μmol/L), n (%)** | **104(22.7)** | **126(27.1)** | **0.008** | **121(22.6)** | **109(28.1)** | **0.019** |

Values are median (interquartile range) or n (%)

**Abbreviations:** SBP, Systolic blood pressure; DBP, diastolic blood pressure; NIHSS, National Institutes of Health Stroke Scale; CHD, Coronary artery disease; FPG, Fasting plasma glucose ; eGFR, estimated glomerular filtration rate; HDL-C, high density lipoprotein cholesterol; LDL-C, low density lipoprotein cholesterol; tHcy, total homocysteine; ARWMC, Age-Related White Matter Changes.

**Table B. Univariate analysis for predictors of WMH locations according to the Fazekas scale**

|  | **PWMHs** | | | **DWMHs** | | |
| --- | --- | --- | --- | --- | --- | --- |
|  | **Mild** | **Severe** | **p-Value** | **Mild** | **severe** | **p-Value** |
| **Sex(female), n (%)** | **152(30.8)** | **140(32.6)** | **0.574** | **238(30.6)** | **54(37.2)** | **0.114** |
| **Age(years)** | **56(47, 63)** | **63(57, 70)** | **<0.001** | **59(50, 66)** | **65(58, 71)** | **<0.001** |
| **SBP(mmHg)** | **139(128, 150)** | **142(130, 156)** | **0.002** | **140(128, 153)** | **143(135, 157)** | **0.006** |
| **DBP(mmHg)** | **85(77, 93)** | **85(78, 90)** | **0.665** | **85(78, 92)** | **86(80, 90)** | **0.261** |
| **NIHSS score( points)** | **3(1, 6)** | **3(2, 6)** | **0.112** | **3(2, 5)** | **3(2, 5)** | **0.620** |
| **Tobacco use, n (%)** | **153(31.0)** | **118(27.4)** | **0.232** | **233(29.9)** | **38(26.2)** | **0.364** |
| **Alcohol use, n (%)** | **121(24.5)** | **91(21.2)** | **0.223** | **188(24.2)** | **24(16.6)** | **0.046** |
| **Hypertension, n (%)** | **250(50.7)** | **299(69.5)** | **<0.001** | **450(57.8)** | **99(68.3)** | **0.019** |
| **Prior stroke, n (%)** | **80(16.2)** | **146(34.0)** | **<0.001** | **177(22.8)** | **49(33.8)** | **0.005** |
| **CHD, n (%)** | **45(9.1)** | **49(11.4)** | **0.256** | **74(9.5)** | **20(13.8)** | **0.118** |
| **Atrial fibrillation, n (%)** | **10(2.0)** | **9(2.1)** | **0.945** | **16(2.1)** | **3(2.1)** | **0.992** |
| **Diabetes mellitus, n (%)** | **110(22.3)** | **89(20.7)** | **0.552** | **174(22.4)** | **25(17.2)** | **0.168** |
| **Dyslipidemia, n (%)** | **38(7.7)** | **27(6.3)** | **0.397** | **58(7.5)** | **7(4.8)** | **0.256** |
| **Haemoglobin(g/L)** | **137(127, 146)** | **134(125, 143)** | **0.006** | **136(126, 146)** | **133(124, 141)** | **0.024** |
| **Hematocrit(%)** | **41.2(38.4, 44.0)** | **40.6(37.6, 43.2)** | **0.013** | **41.0(38.0, 44.0)** | **40.3(37.6, 43.4)** | **0.202** |
| **FPG(mmol/L)** | **5.2(4.6, 6.5)** | **5.2(4.7, 6.4)** | **0.719** | **5.2(4.6, 6.5)** | **5.0(4.6, 5.9)** | **0.023** |
| **Creatinine(μmol/L)** | **66(56, 78)** | **68(57, 78)** | **0.082** | **67(56, 78)** | **68(58, 77)** | **0.536** |
| **eGFR(ml/min/1.73 m²)** | **108.0(92.6, 128.9)** | **101.7(86.5, 115.6)** | **<0.001** | **106.5(90.4, 124.9)** | **100.5(87.1, 112.4)** | **0.004** |
| **Uric acid(μmol/L)** | **279(225, 330)** | **272(215, 327)** | **0.140** | **275(218, 328)** | **278(221, 342)** | **0.455** |
| **Triglyceride (mmol/L)** | **1.5(1.0, 2.2)** | **1.3(1.0, 1.8)** | **<0.001** | **1.4(1.0, 2.1)** | **1.4(1.0, 2.0)** | **0.800** |
| **Total cholesterol (mmol/L)** | **4.2(3.6, 4.9)** | **4.1(3.6, 4.9)** | **0.412** | **4.2(3.6, 4.9)** | **4.1(3.6, 4.8)** | **0.547** |
| **HDL-C(mmol/L)** | **1.0(0.9, 1.2)** | **1.1(0.9, 1.3)** | **0.007** | **1.0(0.9, 1.2)** | **1.1(0.9, 1.3)** | **0.293** |
| **LDL-C(mmol/L)** | **2.6(2.1, 3.3)** | **2.6(2.1, 3.2)** | **0.495** | **2.6(2.1, 3.3)** | **2.6(2.0, 3.1)** | **0.136** |
| **tHcy(>22.13μmol/L), n (%)** | **113(22.9)** | **117(27.2)** | **0.007** | **194(24.9)** | **36(24.8)** | **0.100** |

Values are median (interquartile range) or n (%)

**Abbreviations:** SBP, Systolic blood pressure; DBP, diastolic blood pressure; NIHSS, National Institutes of Health Stroke Scale; CHD, Coronary artery disease; FPG, Fasting plasma glucose ; eGFR, estimated glomerular filtration rate; HDL-C, high density lipoprotein cholesterol; LDL-C, low density lipoprotein cholesterol; tHcy, total homocysteine; ARWMC, Age-Related White Matter Changes.

**Table C. Univariate analysis for predictors of the frontal WMH according to the ARWMC scale**

|  | **left frontal area** | | | **right frontal area** | | |
| --- | --- | --- | --- | --- | --- | --- |
|  | **mild** | **Severe** | **p-Value** | **Mild** | **severe** | **p-Value** |
| **Sex(female), n (%)** | **201(30.5)** | **91(34.3)** | **0.263** | **209(31.1)** | **83(32.9)** | **0.603** |
| **Age(years)** | **57(49, 64)** | **65(59, 73)** | **<0.001** | **58(49, 64)** | **65(58, 73)** | **<0.001** |
| **SBP(mmHg)** | **139(128, 150)** | **145(134, 160)** | **<0.001** | **140(128, 152)** | **142(133, 158)** | **0.001** |
| **DBP(mmHg)** | **84(77, 92)** | **87(80, 92)** | **0.024** | **84(77, 92)** | **87(79, 90)** | **0.334** |
| **NIHSS score( points)** | **3(1, 5)** | **3(2, 6)** | **0.353** | **3(1, 5)** | **3(2, 6)** | **0.359** |
| **Tobacco use, n (%)** | **172(33.0)** | **195(34.0)** | **0.705** | **206(30.7)** | **65(25.8)** | **0.145** |
| **Alcohol use, n (%)** | **131(25.1)** | **152(26.5)** | **0.589** | **161(24.0)** | **51(20.2)** | **0.227** |
| **Hypertension, n (%)** | **261(50.0)** | **387(67.5)** | **<0.001** | **373(55.6)** | **176(69.8)** | **<0.001** |
| **Prior stroke, n (%)** | **89(17.0)** | **182(31.8)** | **<0.001** | **137(20.4)** | **89(35.3)** | **<0.001** |
| **CHD, n (%)** | **42(8.0)** | **66(11.5)** | **0.054** | **65(9.7)** | **29(11.5)** | **0.415** |
| **Atrial fibrillation, n (%)** | **10(1.9)** | **15(2.6)** | **0.437** | **15(2.2)** | **4(1.6)** | **0.537** |
| **Diabetes mellitus, n (%)** | **122(23.4)** | **141(24.6)** | **0.633** | **149(22.2)** | **50(19.8)** | **0.437** |
| **Dyslipidemia, n (%)** | **47(9.0)** | **45(7.9)** | **0.493** | **53(7.9)** | **12(4.8)** | **0.097** |
| **Haemoglobin(g/L)** | **137(126, 146)** | **134(125, 144)** | **0.015** | **136(126, 146)** | **134(124, 143)** | **0.017** |
| **Hematocrit(%)** | **41(38, 44)** | **41(38, 43)** | **0.078** | **41(38, 44)** | **41(37, 44)** | **0.129** |
| **FPG(mmol/L)** | **5.2(4.6, 6.4)** | **5.2(4.7, 6.4)** | **0.805** | **5.2(4.6, 6.4)** | **5.1(4.7, 6.4)** | **0.850** |
| **Creatinine(μmol/L)** | **66(57, 77)** | **69(57, 79)** | **0.054** | **66(56, 77)** | **69(58, 80)** | **0.020** |
| **eGFR(ml/min/1.73 m²)** | **107.6(91.7, 126.3)** | **100.5(84.4, 114.9)** | **<0.001** | **107.3(91.5, 125.8)** | **101.1(83.8, 114.8)** | **<0.001** |
| **Uric acid(μmol/L)** | **274(218, 327)** | **277(221, 335)** | **0.375** | **272(217, 328)** | **282(227, 339)** | **0.152** |
| **Triglyceride (mmol/L)** | **1.4(1.0, 2.1)** | **1.3(1.0, 1.8)** | **0.017** | **1.4(1.0, 2.1)** | **1.3(1.0, 1.8)** | **0.075** |
| **Total cholesterol (mmol/L)** | **4.2(3.6, 4.9)** | **4.2(3.6, 4.8)** | **0.414** | **4.3(3.6, 5.0)** | **4.1(3.4, 4.8)** | **0.027** |
| **HDL-C(mmol/L)** | **1.0(0.9, 1.2)** | **1.1(0.9, 1.3)** | **0.168** | **1.0(0.9, 1.3)** | **1.0(0.9, 1.2)** | **0.731** |
| **LDL-C(mmol/L)** | **2.6(2.1, 3.3)** | **2.6(2.1, 3.1)** | **0.228** | **2.6(2.1, 3.3)** | **2.6(2.0, 3.1)** | **0.011** |
| **tHcy(>22.13μmol/L), n (%)** | **150(22.8)** | **80(30.2)** | **0.013** | **155(23.1)** | **75(29.8)** | **0.014** |

Values are median (interquartile range) or n (%)

**Abbreviations:** SBP, Systolic blood pressure; DBP, diastolic blood pressure; NIHSS, National Institutes of Health Stroke Scale; CHD, Coronary artery disease; FPG, Fasting plasma glucose ; eGFR, estimated glomerular filtration rate; HDL-C, high density lipoprotein cholesterol; LDL-C, low density lipoprotein cholesterol; tHcy, total homocysteine; ARWMC, Age-Related White Matter Changes.
